# Packaging, Purification, and Titration of Replication-Deficient Semliki Forest Virus-Derived Particles as a Self-Amplifying mRNA Vaccine Vector

**DOI:** 10.52547/ibj.3535

**Published:** 2022-04-26

**Authors:** Nastaran Sadat Savar, Thomas Vallet, Arash Arashkia, Kenneth Lundstrom, Marco Vignuzzi, Hamid Mahmoudzadeh Niknam

**Affiliations:** 1 Immunology Department, Pasteur Institute of Iran, Tehran, Iran;; 2 Institut Pasteur, Viral Populations and Pathogenesis Unit, Centre National de la Recherche Scientifique UMR 3569, Paris, France;; 3 Virology Department, Pasteur Institute of Iran, Tehran, Iran;; 4 PanTherapeutics, CH 1095 Lutry, Switzerland

**Keywords:** mRNA vaccines, Semliki Forest virus, Vaccines

## Abstract

**Background:**

Self-amplifying mRNA is the next-generation vaccine platform with the potential advantages in efficacy and speed of development against infectious diseases and cancer. The main aim was to present optimized and rapid methods for SFV-PD SAM preparation, its packaging, and titer determination. These protocols are provided for producing and harvesting the high yields of VRP-packaged SAM for vaccine studies.

**Methods:**

pSFV-PD-EGFP plasmid was linearized and subjected to *in vitro *transcription. Different concentrations of SFV-PD SAM were first transfected into HEK-293 and BHK-21 cell lines, and EGFP expression at different time points was evaluated by fluorescent microscopy. Replicon particle packaging was achieved by co-transfection of SFV-PD SAM and pSFV-Helper2 RNA into BHK-21 cells. The VRPs were concentrated using ultrafiltration with 100 kDa cut-off. The titers of replicon particles were determined by RT-qPCR.

**Results:**

*In vitro* transcribed SAM encoding EGFP was successfully transfected and expressed in HEK-293 and BHK-21 cell lines. Higher levels of EGFP expression was observed in BHK-21 compared to HEK-293 cells showing more stable protein overexpression and VRP packaging. Using ultrafiltration, the high yields of purified SFV-PD-EGFP particles were rapidly obtained with only minor loss of replicon particles. Accurate and rapid titer determination of replication-deficient particles was achieved by RT-qPCR.

**Conclusion:**

Using optimized methods for SAM transfection, VRP packaging, and concentration, high yields of SFV-PD VRPs could be produced and purified. The RT-qPCR demonstrated to be an accurate and rapid method for titer determination of replication deficient VRPs.

## INTRODUCTION

Self-amplifying mRNA vaccines based on alphaviruses represent the next generation nucleic acid-type platform, which has been developed for targeting both infectious diseases and different cancers^ [1,2]^. While several licensed vaccines are built on the first (live attenuated, killed) and the second generation (subunit) platforms, it is not always practical to use these platforms due to the challenges in vaccine preparation and formulation, safety concerns, and lack of effectiveness. For these reasons, nucleic acid-based vaccines are being developed^[3]^. 

The SAM platform can be considered safer and more effective than conventional plasmid DNA vaccines, excluding the risk of DNA integration into the host genome^[1^^]^. SFV is an enveloped positive-strand RNA virus, a member of the alphavirus genus, whose genome encodes four non-structural genes (nsP1-4)^[^^4^^]^. The nsP4 is the core RNA-dependent RNA polymerase. However, all four nsPs are required for RNA replication in host cells^[4]^ by using genomic RNA as a template to synthesize negative-strand RNA to amplify the viral genome^[5]^. In the SFV expression vector, the viral structural genes have been deleted and replaced by the gene of interest, whose transcription is regulated by the sub-genomic 26S promoter^[6]^. Accordingly, the SFV-based SAM can be transcribed *in vitro* from an SFV plasmid encoding the gene of interest and transfected into cells for translation^[7]^. Previous studies have shown successful high-level expression of heterologous proteins in mammalian cells using SFV-based vectors^[8,9]^. Recombinant protein expression using alphavirus platform is transient due to RNA degradation and apoptosis induced in infected cells^[10]^. However, some SFV vectors, such as the SFV-PD vector containing two mutations in the nsP2 gene, show reduced levels of cytotoxicity^[10]^. Packaging of SFV with pSFV-helper renders replication-deficient particles, which generate one cycle of infection and no additional virus progeny production^[7,11]^. To further enhance the biosafety of the system, the second-generation helper vector (pSFV-Helper2) produces conditionally infectious VRPs, eliminating the propagation of replication-proficient particles^[7,11]^. Following the transfection of the *in vitro* transcribed SFV-PD and Helper2 RNAs, self-amplification of RNA occurs in the host cell, generating high titers of replication-deficient SFV particles^[12,13]^. SAM viral delivery has been used for high level exogenous protein expression in a broad spectrum of hosts^[14]^. Preclinical and clinical applications of SAM viral vectors have proven to be efficient for vaccine development^[13^^]^. It has also been shown that recombinant SFV particles can be developed as prophylactic and therapeutic vaccine platforms against infectious diseases and various cancers since SFV-based antigen expression can induce humoral and cell-mediated immunity and have demonstrated protection against challenges with pathogens and tumor cells in animal models^[14-16]^.This study presents the optimized methods for preparation, transfection, and RT-qPCR titer determination of replication-deficient SFV-PD particles as an alphavirus delivery system for SAM-based vaccines. The main aim of this study was to overcome some of the limitations of conventional and current approaches. The coding sequence of EGFP was cloned into the SFV-PD vector and used as a reporter gene to visualize transfection and transduction events and to facilitate the evaluation of transfection/ transduction efficacy and recombinant protein expression in mammalian cell lines.

## MATERIALS AND METHODS

**SAM preparation**

The EGFP sequence (GenBank: QUH22256.1) was subcloned into the *Bam*HI/*Apa*I site of the pSFV-PD vector ^[10]^. The subcloning procedure was confirmed by restriction mapping and DNA sequencing. The pSFV-PD-EGFP plasmid and pSFV-Helper2 plasmid encoding the structural proteins of SFV were transformed into DH5α competent cells, and the transformants were cultivated in Luria-Bertani (Sigma, UK) broth overnight. The pSFV-PD-EGFP plasmid DNA was then purified using the ZymoPURE™ II Plasmid Midiprep Kit (ZYMO RESEARCH, USA). The plasmid concentration was quantified using a NanoDrop UV-Vis Spectrophotometer (Thermo Fisher Scientific, USA). The quality of extracted DNA was verified by 1% agarose gel electrophoresis. The pSFV-PD-EGFP and pSFV-Helper2 plasmid constructs were linearized by *Nru*1 and *Spe*1 restriction enzymes, respectively and purified using the MN kit PCR product and gel purification kit (MACHEREY NAGEL, Germany). Linearized DNA templates were used for *in vitro* transcription using the mMESSAGE mMACHINE™ SP6 kit (Thermo Fisher Scientific, USA), treated with DNase and purified with the RNeasy clean-up kit (Qiagen, USA). Purified RNA was then quantified using the Qubit RNA quantification system (Thermo Fisher Scientific, USA). The quality of *in vitro* transcribed RNA was evaluated on 1% RNase- and DNase-free agarose gels.

**SAM transfection into HEK-293 and BHK-21** **cell lines **

HEK-293 (ATCC^®^ CRL-1573^™^) and BHK-21 (ATCC^®^ CCL-10^™^) cells were cultured to 90% confluency in DMEM supplemented with 10% FBS, 100 U/mL penicillin, and 100 mg/mL streptomycin (GIBCO-BRL, Germany) in 5% CO_2_ at 37 °C. One day before transfection, 1 × 10^5^ cells were cultured in 24-well plates to reach 85-90% confluency at the time of transfection. Before transfection, the medium was replaced by the fresh medium containing antibiotics and 2% FBS. Different concentrations (0.5, 1, and 2 µg) of *in vitro*-transcribed RNA of SFV-PD-EGFP were transfected into the cells by the TransIT mRNA transfection system (Mirus Bio, USA). These cells were incubated at 37 °C in a CO_2_ incubator for 24 h. The EGFP expression in cells was verified by fluorescent microscopy at 24, 48, and 72 h post transfection. 

**Cell infection with different dilutions of EGFP expressing replicon particles**

BHK-21 cells were seeded in six-well plates (4 × 10^5 ^cells/well) in DMEM medium containing 10% FBS and 1% penicillin/streptomycin. Cells were grown to 85% confluency overnight. The next day, the medium was replaced with a fresh medium (DMEM, 2% FBS), and a mixture of pSFV-Helper2 (0.5 µg/ml)/pSFV-PD-EGFP (1 µg/ml) RNA was transfected into the cells, and 48 hours later, the medium was collected. The conditionally infectious SFV particles^[11]^ were treated with α-chymotrypsin to a final concentration of 500 µg/ml, incubated for 30 min and inactivated with aprotinin (250 µg/ml)^[16]^. Five-fold serial dilutions^[17]^ of replicon particles were then prepared. Cells were pre-seeded and cultured overnight to reach 85% confluency. The following day, the medium was replaced with a fresh medium (DMEM, 5% FBS), and BHK-21 cells were infected with five-fold dilutions of SFV-PD-EGFP replicon particles.

**Harvesting and purification of replicon particles**

A total number of 1.5 × 10^6 ^BHK-21 cells were seeded in a T-75 flask in a volume of 20 mL with complete medium (DMEM + 10% FBS + 1% penicillin/streptomycin) and incubated in 5% CO_2 _at 37°C for 18-24 h,. The cells were washed with the infection medium (containing 2% FBS) once and transfected with 1 µg/ml of *in vitro* transcribed SAM according to the manufacturer's protocol (TransIT mRNA transfection kit). Forty-eight hours post transfection, the supernatants were collected and passed through a 0.2-µm filter to remove cell debris. VRP-containing culture supernatants were further clarified by high-speed centrifugation at 12,000 ×g at 4 °C for 30 min^[18]^. Then VRPs were concentrated using the Amicon® Ultra-15 Centrifugal Filter device (100 kDa; Millipore, USA) in a swinging bucket rotor at 1,000 ×g at 4 °C for 30 minutes, in accordance with the manufacturer’s instructions. The VRPs were stored in 200-µl aliquots at -80 °C for future use. For quantitative studies, the packaged RNA was purified using TRI reagent® (Zymo Research, Australia) and Direct-zol™ (Zymo Research, Australia) RNA kit according to the manufacturer's instructions.

**Quantification and Titration of SFV-PD-EGFP replicon particles by real-time PCR**

The concentration of SFV-PD-EGFP SAM packaged in replicon particles was determined by applying real-time PCR using gene-specific primers for the EGFP, the SFV nsP1 region, and previously described nsP2^[19]^, as listed in Table 1. The primers were designed by applying the Primer Quest™ Tool (https://www.idtdna.com/pages/tools/primerquest). everse transcription and amplifications were performed using the Luna^™^ Universal One-Step RT-qPCR Kit (New England Biolabs, USA), 4 µL of mRNA sample, 50 nM of forward and reverse primers in a total reaction volume of 20 µl. The RT-PCR consisted of 10 min at 55 °C, followed by an initial denaturation step at 95 ºC for 1 min, 40 cycles at 95 ºC for 10 s, and extension at 60 ºC for 30 s. Amplification specificity was validated by melting curve analysis generated at the end of each PCR reaction. The experiments were carried out twice and in triplicates for each data point to verify the consistency of the results. The standard curve for quantitation was obtained by 10-fold serial dilutions (10-0.001 ng) of *in vitro* transcribed SFV-PD-EGFP-SAM. Two independent runs with three replicates of each standard dilution were performed. The standard curve was defined as the regression line of the *in vitro *transcribed SAM concentrations versus Ct. The concentration of the SFV-PD SAM packaged in the replicon particles was calculated based on the obtained standard curve. The total copy number of the replicon particles was calculated by converting the ssRNA mass to copy number using the NEBio Calculator (https://nebiocalculator.neb.com/#!/ ssrnaamt). Each SFV RNA copy was considered to belong to one potentially infectious particle; therefore, virus titers were expressed as virus particles per milliliter (VP/mL).

**Table 1:** List of primers used in this study

**Gene**	**Forward**	**Reverse**
*EGFP*	5’ GAAGTTCATCTGCACCACC 3’	5’ TTGTACTCCAGCTTGTGCC 3’
*nsP1*	5’ ACCAGACACCCAGACAATAG 3’	5’ TCCAACCTCTCCTTCTCCTC 3’
*nsP2*	5’ ACAGACTGTCACTGAGCAG 3’	5’ GTGACCATCTACTGCAGAGA 3’

**Statistical analysis**

Initial data analyses (comparison of mean values and transformation of values) were performed using Microsoft Excel 2019 (Microsoft Germany GmbH, Munich, Germany). GraphPad Prism 8.4 (GraphPad Software Inc., San Diego, CA, USA) was used for further statistical analyses and graph creation. Statistically significant differences were investigated by unpaired t-test. *p* ≤ 0.05 was considered as statistically significant..

## RESULTS

**Gene cloning and SAM preparation**

**Fig 1:**
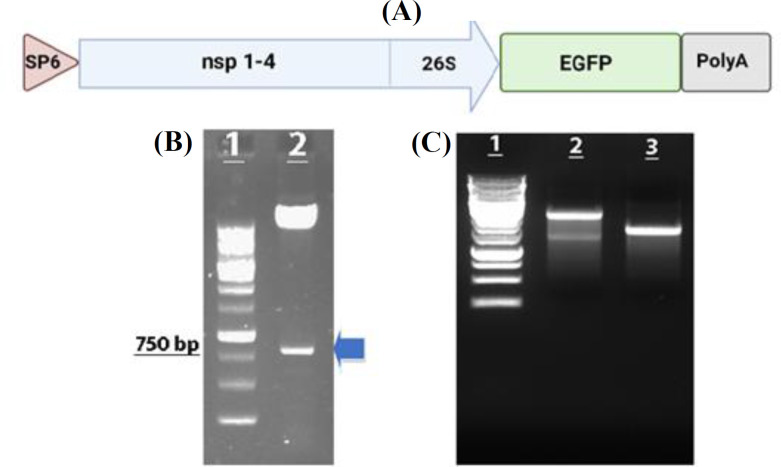
(A) Schematic presentation of SFV-PD vector encoding EGFP reporter. B. Double digestion of recombinant pSFV-PD-EGFP using BamHI and ApaI restriction enzymes; lane 1, ladder (1 kb); lane 2, digested recombinant vector releasing the 750 bp fragment (indicated by the blue arrow). (C) Quality evaluation of in vitro transcribed pSFV-PD-EGFP and Helper2 RNAs; lane 1, 1-kb ladder; lane 2, pSFV-PD-EGFP RNA; lane 3: Helper2 RNA.

The coding sequence of the EGFP gene was subcloned into the pSFV-PD plasmid (~11.2 kb). Figure 1A illustrates a schematic presentation of the pSFV-PD plasmid with the EGFP insert. The subcloning of the EGFP gene was confirmed by double digestion with *Bam*HI and *Apa*I enzymes releasing a fragment of 750 bp (Fig. 1B). Linearized pSFV-PD plasmid encoding the EGFP gene and the pSFV-Helper2 plasmid were used as templates for *in vitro* transcription. The quality of the transcribed SFV-PD-EGFP and Helper2 RNAs was evaluated on 1% agarose gels (Fig. 1C). The quantity of *in vitro *transcribed RNAs was approximately 2 μg/μl.

**SAM transfection into HEK-293 cell line**

**Fig. 2:**
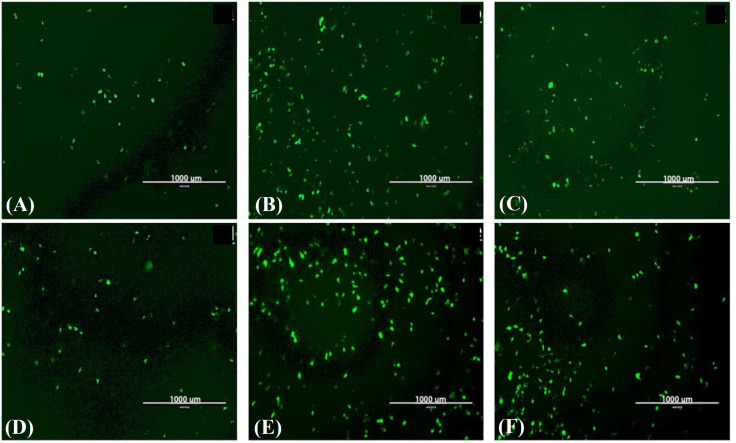
SFV-PD-EGFP SAM transfection into HEK-293. (a-c) Twenty-four hours after transfection with 0.5, 1, and 2 µg RNA, respectively; (d-f) forty-eight hours post transfection with 0.5, 1, and 2 µg RNA, respectively.

Based on fluorescence microscopy, doubling the amount of RNA used for transfection from 0.5 to 1 µg leads to a higher level of EGFP expression. However, there was no difference in transfection rates between 1 and 2 µg SAM at any time point (Fig. 2A-2C). Additionally, 48 hours after transfection, the expression level increased slightly at all concentrations (Fig. 2D-2F). Cell incubation for 72 hours resulted in a high rate of cell death, causing a dramatic decrease in the number of the EGFP expressing cells (data not shown). Although expression of EGFP was confirmed in HEK-293 cells, due to the rapid detachment and a high rate of cell death, transfection and expression potentials in BHK-21 cells were evaluated as an alternative. 

**SAM Transfection into BHK-21 cells**

**Fig. 3:**
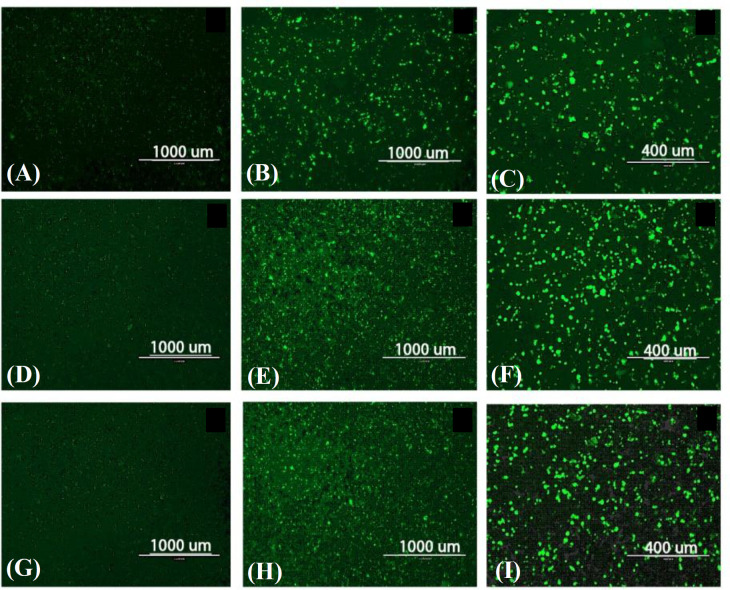
SFV-PD-EGFP SAM transfection into BHK-21 cells. (a-c) Transfection with 0.5 µg SAM at 24 h (a), 48 h (b) and 72 h (c) post transfection; (d-f) transfection with 1 µg SAM at 24 h (d), 48 h (e) and 72 h (f) post transfection; (g-i) transfection with 2 µg SAM at 24 h (g), 48h (h) and 72 h (i) post transfection.

Based on fluorescent microscopy, the transfection rate in BHK-21 cells was significantly higher than that seen in the HEK-293 cell line. The highest rates were obtained with 1 or 2 µg of RNA (Fig. 3). Our data also showed that BHK-21 cells were more stable than HEK-293 cells. Additionally, the highest level of EGFP expression was observed at 72 hours post infection at all concentrations.

**Replicon particle harvesting, purification, and cell infections**

**Fig. 4:**
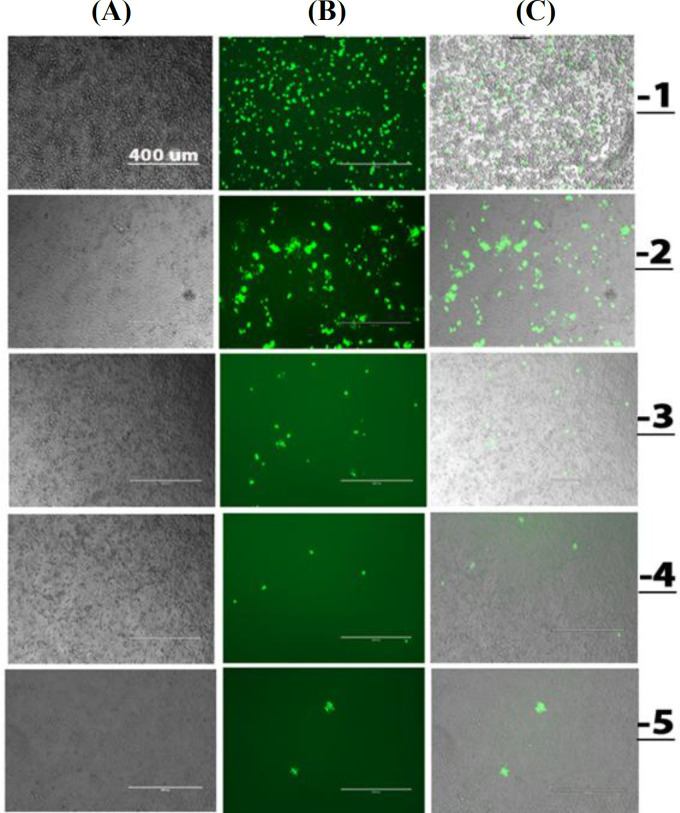
Cell infection with five-fold dilutions of replicon particles. Fluorescence microscopy was performed 48 hours post-infection. Column A, transmission light microscopy; column B, fluorescence microscopy; column C, overlay of transmission light and fluorescence microscopy.

**Fig. 5:**
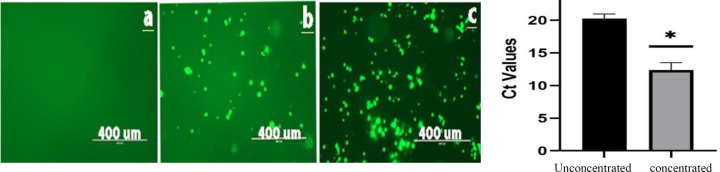
Qualitative and quantitative analyses of replicon particle purification using ultrafiltration. (A) Confirmation of replicon particle purification and concentration in BHK-21 cells 72 hours post infection. a. Cells infected with the flowthrough from the Amicon® Ultra-15 Centrifugal Filter device (100 kDa); (b) cells infected with SFV-PD-EGFP SAM replicon particles (dilution -2) before purification; (c) cells infected with purified and concentrated SFV-PD- EGFP replicon particles (dilution -2). (B) RT-qPCR results for concentrated and unconcentrated replicon particles showing a significantly lower Ct value in the concentrated sample (p ≤ 0.005).

BHK-21 cells were first transfected by *in vitro* transcribed SAM along with pSFV-Helper2 RNA Replicon particles were harvested 72 hours post transfection, activated by α-chymotrypsin treatment and were subjected to the infection of cells using five-fold serially diluted replicon particles, as shown in Figure 4. Based on fluorescence microscopy, dilution-1 indicated the highest number of EGFP positive BHK-21 cells and the highest rate of cell death 48 hours post infection. While infection with dilution -2 generated fewer EGFP positive cells than dilution -1, the cell viability was higher even at 72 hours post infection. The effect of purification and concentration of replicon particles was evaluated in BHK-21 cells infected with the SFV-PD-EGFP dilution-2. Based on fluorescence microscopy, no replicon particles were detected in the flowthrough from the Amicon® Ultra-15 Centrifugal Filter device (100 kDa). Not surprisingly, more infected cells were observed using purified and concentrated replicon particles (Fig. 5A). The difference in amounts of EGFP RNA between concentrated and unconcentrated particles was also verified by RT-qPCR using EGFP primers. According to the results, a significantly higher level of EGFP RNA (lower Ct values) was observed in concentrated samples (*p* ≤ 0.005; Fig. 5B).

**Titration of SFV-PD-EGFP replicon particles by real-time PCR**

**Fig. 6:**
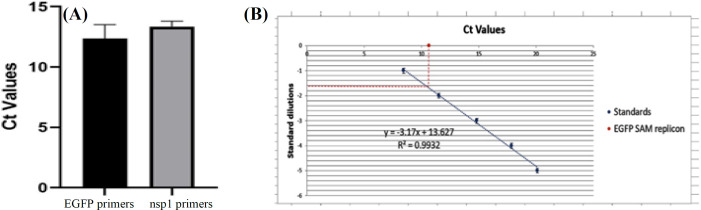
RT-qPCR for titration of packaged replicon particles. (A) Obtained Ct values using EGFP and nsP1 primers for SFV-PD-EGFP SAM. (B) RT-qPCR standard curve for the titration of SFV-PD-EGFP SAM using nsP1 primers.

The purified SFV-PD EGFP SAM obtained from concentrated replicon particles was subjected to RT-qPCR. SFV-PD-EGFP SAM presented a single peak in the melting curve of EGFP primers, which indicated the absence of primer-dimer formation during the reaction and specificity of the amplification^[20]^. The amplification specificity was validated by melting curve analysis generated at the end of each PCR reaction. Our results showed a nonspecific attachment of the reverse primer to the vector and off-target amplification products using previously described nsP2 primers (Table 2), which was further verified by the presence of multiple peaks in the melt curve (Table 2). Consequently, nsP1-specific primers were selected for SFV-PD replicon titration. The primers were then verified using Oligo7 software and real-time PCR (Table 2). The melt curve of the primers showed a single peak at Tm = 85.1, verifying the absence of primer dimer and off-target products (Table 2). Comparison between obtained Ct values using nsP1 and EGFP gene-specific primers revealed no significant difference (Fig. 6A), meaning that the designed nsP1 primers can be used to titrate any recombinant SFV-PD VRPs regardless of the gene of interest. The standard curve for SFV-RNA quantitation was obtained from five 10-fold dilutions (10-0.001 ng) of *in vitro *transcribed pSFV-PD-EGFP SAM. The mean Cts corresponding to each logarithm of the standard copy number was plotted. The slope of the regression line was -3.17, and the correlation coefﬁcient R^2^ was 0.993 (Fig. 6B). The amplification efficiency was calculated above 2 using the E=10^(-1/slope) ^formula^[20]^. The concentration of SFV-PD-EGFP SAM packaged in viral particles was obtained based on the standard curve as indicated in Figure 6B. The total copy number of replicon particles was then calculated by converting the ssRNA mass to copy number using the NEBio Calculator. According to the results, 1.6 ng/4 µl of a 12-kb SFV-PD-EGFP SAM equals to 6.2 × 10^7 ^ssRNA copies or replicon particles/ µl. The total yield of concentrated replicon particles was calculated as 6.2 × 10^10 ^VRP/ml.

## DISCUSSION

 SAM vaccines possess the ﬂexibility of plasmid DNA vaccines with enhanced immunogenicity and safety^[3]^. However, efficient delivery of SAM to the cell cytoplasm is essential for amplification and expression of the encoded antigen of interest^[3]^. Sophisticated mechanisms have evolved for alphavirus RNA delivery to host cells^[22]^. VRP-SAM-based vaccine candidates have been broadly tested against viral, bacterial, and protozoan pathogens and tumor cells in different animal models^[8,9]^. Packaging of SAM in viral particles provides superior delivery and efficiently protects SAM from ribonucleases^[23]^. It has been shown that under *in vitro *and *in vivo* conditions, the VRPs can infect cells and express the encoded gene of interest^[24,25]^. However, as a safety feature, the use of replication-deficient VRPs only allows one round of infection. These suicide particles are incapable of virus replication and production of virus progeny, preventing the spread of the virus^[11]^.

In the present study, EGFP expression was evaluated in HEK-293 cells transfected with different amounts of *in vitro *transcribed SFV-PD-EGFP SAM (Fig. 2). Fluorescence microscopy demonstrated that the rapid detachment and a high rate of cell death occurred 48 hours post transfection, suggesting that HEK-293 may not be suitable for replicon particle packaging and production. This incidence could be due to limitations in protein overexpression and vesicular trafficking^[26]^. It has been displayed that following transfection, protein overexpression can cause cellular defects in many cell lines, including HEK-293^[26]^. Consequently, the lower limitations of EGFP expression in BHK-21 cells lead to the higher levels of prolonged EGFP expression (Fig. 3). Therefore, the BHK-21 cell line was indicated to be more suitable for further VRP packaging and expression studies. 

 Infection of BHK-21 cells with serial dilutions of SFV-PD-EGFP replicon particles indicated the highest level of cell infection for the smallest dilution (dilution -1; Figure 4. However, the highest level of cytotoxicity was also seen for this dilution. Accordingly, appropriate dilutions could be applied depending on the time point for optimal antigen expression and the rate of protein degradation in the host cells^[27]^. As shown a dramatic decrease in growth, infected cells could be easily distinguished from noninfected control cells through their rounded morphology (Fig. 4). This observation was consistent with previous reports on cell morphological changes following alphavirus infections^[28]^. 

The concentration of viral particles through ultra-centrifugation on sucrose gradients is a commonly used technique for enveloped viruses^[29,30]^. However, this method was less efficient than ultracentrifugation through a 20% and 50% double iodixanol cushion for alphaviruses and provided only a four-fold virus concentration^[31]^. Moreover, both sucrose and iodixanol cushion methods are labor-intensive and time-consuming, making the technique described here attractive as it is efficient and rapid and does not require ultracentrifugation. This approach enabled the concentration of SFV-PD VRPs up to 100 times (Fig. 5A and 5B). Comparison of Ct values before and after concentration allowed estimating the efficacy of the proposed method (Fig. 5B). However, due to virus aggregation, too high concentration of the virus is not recommended.

**Table 2:** Comparison between previously introduced nsP2 (19) and the designed (nsP1) primers for SFV-PD titration

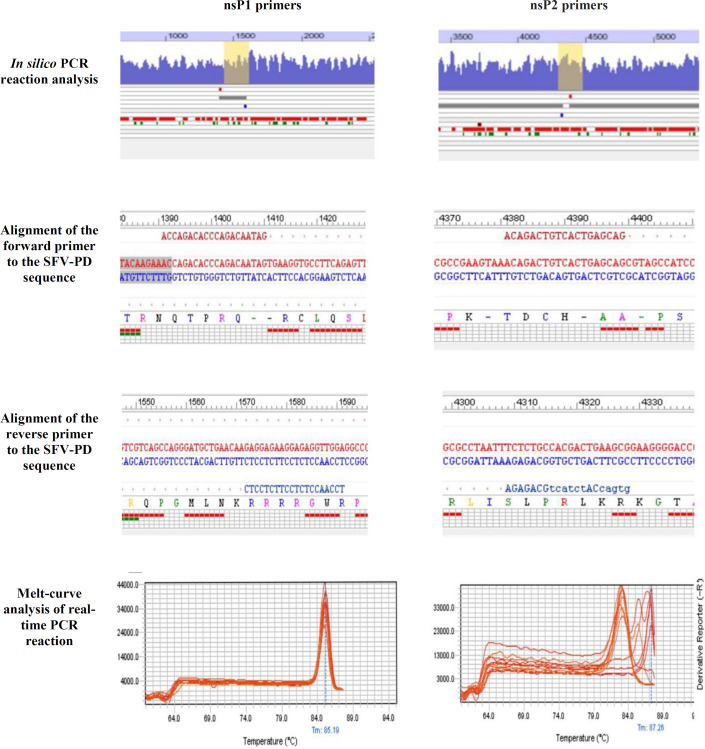

Application of ultrafiltration with a cut-off of 100 kDa has previously been reported for concentrating lentiviruses from cell culture supernatant, which resulted in high recovery of infectious particles^[32]^. However, to the best of our knowledge, this is the first application of this method for alphavirus purification. The key to accurate titer determination of replication-deficient SFV-PD particles is to find an alternative for commonly used methods for virus-based plaque assays and TCID_50_, which are restricted to replication-proficient particles^[33]^. Additionally, titer determinations based on reporter gene expression (EGFP, β-galactosidase) and immunofluorescence have been used for replication-proficient and -deficient viruses^[17,34]^, but they are not very accurate for titer determination. Formerly, a standard titer determination method has been described for replication-deficient SFV particles based on RT-qPCR using nsP2 primers^[19]^. While it has been claimed that the designed primers could be used for titration of all types of recombinant SFV particles, our results revealed that these primers did not target the nsP2 region of SFV-PD (Table 2). 

In the present study, we described the fast and optimized methods for *in vitro* transcription, transfection, packaging, purification, and titration of SFV-PD SAM VRPs. The purified replicon particles encoding different antigen proteins from a wide range of pathogens can be used for *in vitro* and *in vivo *vaccine studies.

## DECLARATIONS

### Ethical statement

Not applicable.

### Data availability

Data supporting this article are included within the article, and the raw data sets of this study are available from the corresponding authors on reasonable request.

### Author contributions

NSS, conceptualization, methodology, investigation, validation, writing original draft; TV, resources and investigation; AA, methodology and supervision; KL, methodology, writing, review, and editing; MV, HMN, supervision, conceptualization, project administration, and funding acquisition.

### Conflict of interest

None declared.

### Funding/support

To pursue this study, Nastaran Sadat Savar was supported by a Ph.D. scholarship from the Pasteur Institute of Iran (Grant ID: TP-9344). This work was also partly funded by the Agence Nationale de Recherche Laboratoire d’Excellence grant ANR-10-LABX-62-IBEID.
